# Hemodynamic Disturbances in Posterior Circulation Stroke: 4D Flow Magnetic Resonance Imaging Added to Computed Tomography Angiography

**DOI:** 10.3389/fnins.2021.656769

**Published:** 2021-09-30

**Authors:** Jan Malm, Johan Birnefeld, Laleh Zarrinkoob, Anders Wåhlin, Anders Eklund

**Affiliations:** ^1^Department of Clinical Science, Neurosciences, Umeå University, Umeå, Sweden; ^2^Department of Surgical and Perioperative Sciences, Umeå University, Umeå, Sweden; ^3^Department of Radiation Sciences, Umeå University, Umeå, Sweden; ^4^Umeå Centre for Functional Brain Imaging, Umeå University, Umeå, Sweden; ^5^Centre for Biomedical Engineering and Physics, Umeå University, Umeå, Sweden

**Keywords:** blood flow, hemodynamics, brain infarction, magnetic resonance imaging, vertebral artery, basilar artery, posterior cerebral artery, stroke

## Abstract

**Objective:** A clinically feasible, non-invasive method to quantify blood flow, hemodynamics, and collateral flow in the vertebrobasilar arterial tree is missing. The objective of this study was to evaluate the feasibility of quantifying blood flow and blood flow patterns using 4D flow magnetic resonance imaging (MRI) in consecutive patients after an ischemic stroke in the posterior circulation. We also explore if 4D-flow, analyzed in conjunction with computed tomography angiography (CTA), has potential as a diagnostic tool in posterior circulation stroke.

**Methods:** Twenty-five patients (mean age 62 years; eight women) with acute ischemic stroke in the posterior circulation were investigated. At admission, all patients were examined with CTA followed by MRI (4D flow MRI and diffusion-weighted sequences) at median 4 days after the presenting event. Based on the classification of Caplan, patients were divided into proximal/middle (*n* = 16) and distal territory infarcts (*n* = 9). Absolute and relative blood flow rates were calculated for internal carotid arteries (ICA), vertebral arteries (VA), basilar artery (BA), posterior cerebral arteries (P1 and P2), and the posterior communicating arteries (Pcom). In a control group consisting of healthy elderly, the 90th and 10th percentiles of flow were calculated in order to define normal, increased, or decreased blood flow in each artery. “Major hemodynamic disturbance” was defined as low BA flow and either low P2 flow or high Pcom flow. Various minor hemodynamic disturbances were also defined. Blood flow rates were compared between groups. In addition, a comprehensive analysis of each patient’s blood flow profile was performed by assessing relative blood flow rates in each artery in conjunction with findings from CTA.

**Results:** There was no difference in total cerebral blood flow between patients and controls [604 ± 117 ml/min vs. 587 ± 169 ml/min (mean ± SD), *p* = 0.39] or in total inflow to the posterior circulation (i.e., the sum of total VA and Pcom flows, 159 ± 63 ml/min vs. 164 ± 52 ml/min, *p* = 0.98). In individual arteries, there were no significant differences between patients and controls in absolute or relative flow. However, patients had larger interindividual relative flow variance in BA, P1, and P2 (*p* = 0.01, <0.01, and 0.02, respectively). Out of the 16 patients that had proximal/middle territory infarcts, nine had CTA findings in VA and/or BA generating five with major hemodynamic disturbance identified with 4D flow MRI. For those without CTA findings, seven had no or minor 4D flow MRI hemodynamic disturbance. Among nine patients with distal territory infarcts, one had major hemodynamic disturbances, while the remaining had minor disturbances.

**Conclusion:** 4D flow MRI contributed to the identification of the patients who had major hemodynamic disturbances from the vascular pathologies revealed on CTA. We thus conclude that 4D flow MRI could add valuable hemodynamic information when used in conjunction with CTA.

## Introduction

About one-fifth of ischemic stroke affects the posterior circulation (Savitz and Caplan, 2005), and patients with a symptomatic intracranial vertebrobasilar (VB) stenosis have an increased risk for recurrent stroke (Qureshi et al., 2003; Caplan et al., 2004; Gulli et al., 2013). In current clinical practice, vascular imaging is a central part of the evaluation in a patient with VB stroke. Among the most commonly used modalities is the computed tomography angiography (CTA). In a CTA, iodine contrast agent is injected intravenously, and image acquisition is performed in the arterial phase. CTA provides detailed information on vascular anatomy and structural pathology such as stenoses and occlusions. However, it provides little information regarding blood flow other than whether an artery fills with contrast agent or not. Another modality often used in conjunction with CTA is diffusion-weighted magnetic resonance imaging (DW-MRI). It is sensitive to acute ischemia and associated cytotoxic edema, as this causes water to shift from the extracellular- to the intracellular space, thus restricting diffusion (Roberts and Rowley, 2003).

Little is known about quantitative blood flow changes in the VB arteries after a stroke. Hemodynamic disturbances could be created by atherosclerotic stenoses or occlusions, congenital anatomical vascular variants, and physiological factors such as a low blood pressure. The current knowledge about quantitative blood flow disturbances in the VB territory is mostly based on the Vertebral Flow Evaluation and Risk of Transient Ischemic Attack and Stroke (VERiTAS) study. Using phase-contrast MRI, it was found that in patients with symptomatic bilateral vertebral artery (VA) stenosis or occlusion, a worse outcome could be predicted when blood flow in basilar (BA) and posterior cerebral (PCA) arteries was decreased (Amin-Hanjani et al., 2005, 2016). Phase-contrast MRI (2D PCMRI) can be used to quantitatively measure intraarterial blood flow, although it is hampered by the need to scan each measurement plane separately. Thus, it is time consuming and may not be suitable for clinical routine in the context of a comprehensive vascular workup.

A development of 2D PCMRI is 4D flow MRI (Gu et al., 2005; Morgan et al., 2020). It provides the ability to simultaneously measure blood flow rates in all major arteries following a single acquisition (Strater et al., 2018). The technology is promising from a clinical perspective: in post processing, a 3D angiogram is used to locate vessel segments in which the diameter, flow rate, flow velocity, flow direction, and pulsatility can be examined. Clinical implementations have previously been limited by long scan times, but the use of undersampling techniques such as phase-contrast vastly undersampled isotropic projection reconstruction (PC VIPR; Gu et al., 2005) has cut scan times to under 10 min. The main strength of 4D flow MRI, used in conjunction with CTA, is the addition of physiological parameters besides the anatomical imaging. For example, information from CTA regarding a high-grade VA stenosis may be complemented with 4D flow data on whether blood flow is decreased across the stenosis or if there is compensatory flow from the contralateral VA or from the posterior communicating arteries (Pcom). 4D flow MRI has previously mainly been used in cardiac imaging (Strater et al., 2018). In neurology and neurosurgery, there is currently no evidence-based indication for this technique (Morgan et al., 2020). A comprehensive quantitative assessment of the posterior circulation using this modality has not been previously published.

Stenting as secondary stroke prevention has not yet received a major breakthrough due to a high frequency of complications. Relating to this, patient selection procedures seem to be a main obstacle (Compter et al., 2015; Jenkins and Stewart, 2017; Markus et al., 2017; Drazyk and Markus, 2018). The data from VERiTAS suggests that quantitative blood flow measurements could play a role in selecting patients with high risk of stroke recurrence for endovascular procedures in the VB circulation (Amin-Hanjani et al., 2016). Compared to 2D PCMRI used in VERiTAS, the capability of 4D flow MRI to simultaneously measure flow in all cerebral arteries could be a major advantage in the analyses of distribution of blood supply to the posterior circulation.

We thus hypothesized that quantitative blood flow measurements from 4D flow MRI could be used to detect hemodynamic disturbances in patients with VB stroke. Considering this, the objective of this study was to evaluate the feasibility of quantifying blood flow and its patterns using 4D flow MRI in consecutive patients after an ischemic stroke in the posterior circulation and to explore if flow patterns indicating hemodynamic disturbances and a compromised blood flow identified with 4D flow MRI and analyzed in conjunction with CTA and DW-MRI have potential as a future diagnostic tool in posterior circulation stroke.

## Materials and Methods

In summary, this is a prospective study with consecutive recruitment of patients with brain infarction in the posterior circulation. They were examined with brain CT and CTA. This was followed within 1 week by brain MRI, which included DW-MRI and quantitative assessment of flow using 4D flow MRI. Patients and a separately collected control group were compared, and specific blood flow patterns were described on an individual level.

### Patients and Controls

The study population in this prospective feasibility study was consecutive patients with infarction in the posterior circulation admitted to the Stroke Unit at Umeå University between 2012 and 2015. Patients were eligible if they were 18–84 years old and had a clinical diagnosis of brain infarction related to blood supply from the vertebral, basilar, or PCAs. Exclusion criteria included previous stroke ipsilateral to the current event, unilateral or bilateral fetal-type PCA, pre-existing disability (defined as modified Rankin scale > 1), and previous disease of the central nervous system. In addition, patients with any MRI contraindication, systolic blood pressure > 180 mmHg, Mini-mental State Examination < 23 points, or inability to understand oral information and/or provide informed consent were excluded. The MRI scanner was located in a research facility, and the fire emergency preparedness plan did not allow bed-ridden patients to be investigated. In total, 27 patients were enrolled; two had fetal-type PCA and were excluded. Thus, the study population consisted of 25 patients. Clinical features are described in Table 1.

**TABLE 1:** Characteristics of patients and controls.

	Patients *N* = 25 *n* (%)	Controls *N* = 15 *n* (%)	*p*-value
Female	9 (36)	5 (33)	ns
Age (mean ± SD)	63 ± 15	77 ± 4	<0.001
<60 years	9 (39)	0	
tPA treatment	5 (20)	–	
Risk factors			
Hypertension	19 (76)	9 (60)	ns
Hyperlipidemia	22 (88)	7 (47)	<0.01
Atrial fibrillation	0	0	ns
Previous stroke	1 (4)	0	ns
Ischemic heart disease	5 (20)	1 (7)	ns
Smoking (current or previous)	14 (56)	2 (13)	<0.01
Peripheral artery disease	0	0	ns
Diabetes	1 (4)	1 (7)	ns
Blood pressure (BP)			
Systolic BP, mm Hg	144 ± 22	141 ± 16	ns
Diastolic BP, mm Hg	79 ± 12	85 ± 11	ns
Heart rate	67 ± 11	69 ± 16.6	ns

At time of admittance, all patients had a brain CT and CTA. Five patients (20%) were treated with tissue-type plasminogen activator (tPA) in the acute phase. No mechanical thrombectomies were performed. All included patients were examined with MRI (see below for details) at median 4 [interquartile range (IQR), 2–7] days of stroke onset. A history was obtained, and a neurological examination was performed by a stroke specialist. The standardized diagnostic workup included standard blood tests and electrocardiography (performed twice). Long-term Holter ECG monitoring was performed to exclude atrial fibrillation.

A scheme initially introduced by Caplan (1996) was used to divide lesions into three vascular territories (i.e., proximal, middle, and distal territory), based on anatomy and the supply zone of each artery. The proximal territory included VA and posterior inferior cerebellar artery (PICA), the middle territory, the BA up to the superior cerebellar artery branches, and the distal territory the top of the basilar including the PCAs (P1 and P2).

The control group consisted of elderly volunteers that had been examined clinically and with the same 4D flow MRI protocol as the patients. They have been described in detail elsewhere (Dunas et al., 2019). Exclusion criteria were the same as for patients and additionally included any previous cerebrovascular event. 35 subjects were initially recruited; 20 were subsequently excluded because of previous cerebrovascular events (*n* = 5), neurodegenerative disease (*n* = 2), fetal-type PCA (*n* = 12), and technical reasons (*n* = 1). The final control group therefore contained 15 healthy elderly. Characteristics of the controls are described in Table 1. Compared to patients, controls were older, less likely to smoke or have smoked, and less likely to have hyperlipidemia. Heart rate, systolic- and diastolic blood pressure, and frequency of hypertension were similar in patients and controls.

### Ethical Considerations

The study was approved by the ethical review board at Umeå University. Oral and written informed consent according to the Declaration of Helsinki was obtained.

### Magnetic Resonance Imaging Including 4D Flow Magnetic Resonance Imaging

A 3T MRI scanner (GE Discovery MR 750, Waukesha, WI, United States) with a 32-channel head coil was used to obtain standard sequences (T1, fluid-attenuated inversion recovery, and DW-MRI). Flow velocities in arteries were measured with 4D flow MRI and the PC VIPR pulse sequence (Gu et al., 2005), which features three-dimensional radial k-space sampling. Two scans were acquired with velocity encoding (VENC) 110 and 40 cm/s, respectively. The following scanning parameters were used: repetition time/echo time (TR/TE) of 6.5/2.7 ms for the VENC 110 scans and 6.9/3.1 ms for the VENC 40 scan, flip angle 8°, and bandwidth of 166.67 kHz. For each encoding direction, 16,000 radial k-space projections were acquired using an acquisition matrix of 300 × 300 × 300 and an imaging volume of 220 mm × 220 mm × 220 mm. Data were reconstructed into 0.69 mm × 0.69 mm × 0.69 mm voxels. The scan time was approximately 9 min for each VENC.

### Imaging Analysis and Blood Flow Measurements

**FIGURE 1:**
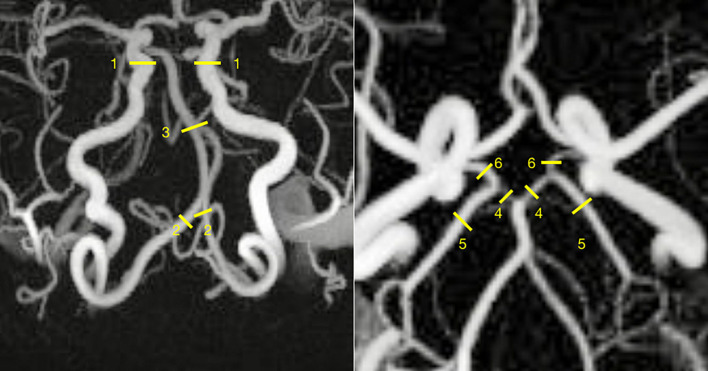
Sample 4D flow MRI complex difference angiogram. Flow was measured in the internal carotid arteries (1), vertebral arteries (2), basilar artery (3), P1 and P2 segments of the posterior cerebral artery (4, 5), and the posterior communicating arteries (6).

Blood flow measurements were made using software developed in-house (Wahlin et al., 2013). A single operator (JB) made all measurements after reviewing CTA findings. First, VENC 40 image data were manually inspected for signs of aliasing. If none were present, all locations were measured using VENC 40. For locations with signs of aliasing, VENC 110 was used. In each artery, flow was measured at three points in close proximity and averaged. Care was taken that the difference between the largest and smallest measurement did not exceed 20% of the largest (Zarrinkoob et al., 2019). If so, a new measuring point was selected. The following measurement points were used: VA (V4 segment, distal to PICA), BA (proximal to anterior inferior cerebellar arteries), PCA (P1 and P2 segments), Pcom (middle), and internal carotid artery (ICA, cavernous segment). Paired arteries were measured bilaterally. Example complex difference angiograms with measuring points are presented in Figure 1. Aliasing in VENC 40 was found in most ICAs (*n* = 77). In the remaining arteries, aliasing in VENC 40 occurred in VA (*n* = 3), BA (*n* = 5), PCA (*n* = 5), and Pcom (*n* = 3) distributed among eight patients and two controls.

In patients and controls, blood flow rate (in ml/min) was calculated for ICA, VA, BA, PCA (P1 and P2 segments), and Pcom. For each patient and every artery, a relative flow rate was thereafter calculated by dividing the blood flow rate by the total cerebral blood flow (tCBF), calculated as the sum of flow in ICA and VA bilaterally (Zarrinkoob et al., 2015). Blood flow rates are presented as percent of tCBF (%tCBF) unless otherwise specified. In order to assess reliability, a second operator (LZ) measured five cases (55 arteries) independently and blinded to CTA findings. The intraclass correlation (ICC, two-way random, single rater, and absolute agreement) was calculated.

### Anatomic Variations

Each subject’s anatomy was assessed for known anatomic variants using CTA. Presence or absence of Pcom was recorded. Fetal anatomy was defined as one or both P1 segments missing or being hypoplastic, the corresponding P2 stemming mainly from Pcom and no explanatory angiographic pathology. Hypoplastic VA was defined as diameter <2 mm in the V4 segment and the diameter ratio to the contralateral side being ≤1:1.7 along the entire length of the artery (Thierfelder et al., 2014).

### Schematic Diagrams

In order to facilitate a comprehensive assessment of each patients flow pattern, an individual schematic flow diagram was created for all patients. This included vascular anatomy, vascular pathology, ischemic lesions, and intraarterial flow. DW-MRI and CTA were assessed by a neuroradiologist, and based on this description, schematic diagrams were created by hand. Any occlusion, dissection, atherosclerotic stenosis ≥50%, or free-floating thrombus as observed on CTA was schematically represented. Lesions with high DW-MRI signal and low apparent diffusion coefficient on MRI were interpreted as acute ischemia and schematically represented. Finally, the relative flow for each artery was presented.

### Definitions of Flow Deviations and Hemodynamic Disturbances

The 90th and 10th percentile of relative flow in controls was used to define normal-, low-, and high flow in each artery. The definition of “major hemodynamic disturbance” was developed from the VERiTAS study (Amin-Hanjani et al., 2015, 2016). Major hemodynamic disturbance was defined as low BA flow in conjunction with either bilateral low P2 flow (indicating low distal flow) or high Pcom flow (indicating the need for compensatory flow from the anterior circulation). “Minor hemodynamic disturbance” was defined as the presence of any of the following in a patient not fulfilling the criteria for major hemodynamic disturbance: “reverse flow,” defined as an opposite flow compared to the expected, that is, for P1 in the direction toward BA and for Pcom in the direction toward the carotid artery; “leptomeningeal collaterals via P2,” considered to exist if there was an increased blood flow in P2 together with normal BA flow; and low flow in P1 or P2.

### Statistical Analysis

Group-level blood flow rates were presented as mean ± SD unless otherwise specified. As the sample size was limited, we opted for non-parametric tests. Differences between two and three groups were compared using Mann–Whitney *U*-test and Kruskal–Wallis test, respectively. To test for equal variances, the Brown–Forsythe test was used. For differences in frequency, the chi-square test was used. Spearman’s rho was used for all reported correlations. The significance level was set as *p* < 0.05.

## Results

Based on DW-MRI, 13 patients in this study had proximal or middle territory infarcts (nine had a corresponding stenosis or occlusion on CTA); nine patients had distal territory infarcts on DW-MRI (four had a corresponding stenosis or occlusion on CTA). Two patients had brainstem infarcts and a third normal DW-MRI, all of them without stenosis/occlusions on CTA. According to location on imaging and symptoms, those three patients were classified as proximal territory infarcts. ICC for flow measurements was 0. 98 [95% CI (0.96, 0.99)], which was considered excellent.

### Total Cerebral Blood Flow and Its Distribution to the Posterior Circulation

There was no difference in tCBF between patients and controls (604 ± 117 ml/min vs. 587 ± 169 ml/min, *p* = 0.39) or in total inflow to the posterior circulation (i.e., the sum of total VA and Pcom flows, 159 ± 63 ml/min vs. 164 ± 52 ml/min, *p* = 0.98). There were no significant differences in absolute flow in any individual measuring point.

### Distribution of Flow in the Main Arteries of the Posterior Circulation

**FIGURE 2:**
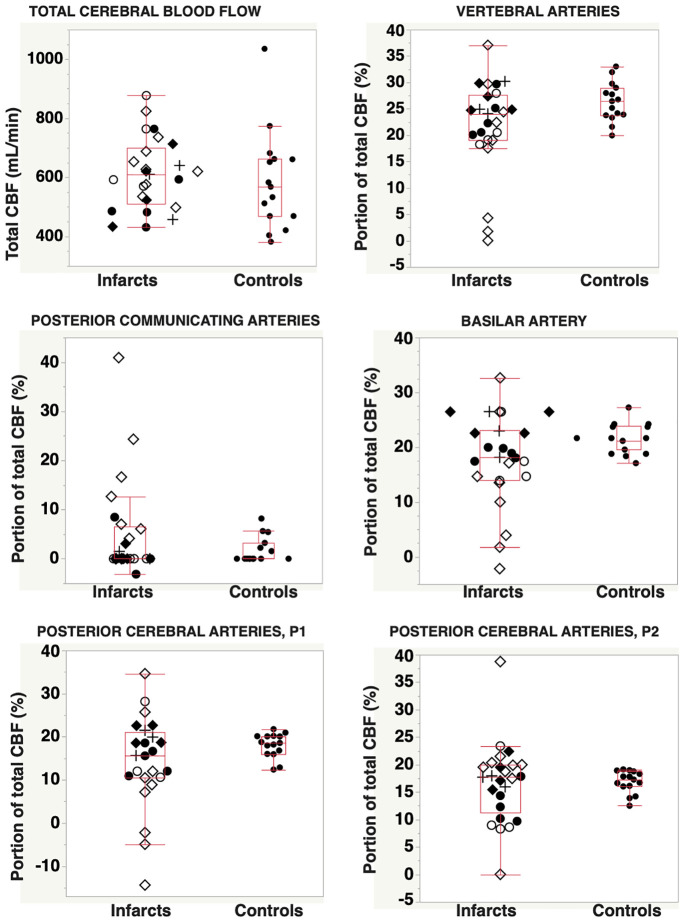
Total cerebral blood flow and distribution of total CBF into the posterior circulation vascular tree, for patients with posterior circulation infarcts (*n* = 25) and for controls (*n* = 15). Abbreviations: ⋄, infarction in the proximal/middle territory and CTA-verified occlusion or stenosis of VA and/or BA; ◆, infarct located in the proximal/middle territory, but without angiography findings in VA or BA; ○, infarction in the distal territory and CTA verified occlusion or stenosis in the PCA; ●, infarct located in the proximal/middle territory, without angiography findings in the PCA; and +, brain stem infarcts classified as proximal/middle territory infarcts and without corresponding CTA findings. CBF, cerebral blood flow; CTA, computed tomography angiography; VA, vertebral arteries; BA, basilar artery; PCA, posterior cerebral artery.

The proportion of flow, expressed as percent of total cerebral blood flow (%tCBF), for each artery of the posterior circulation, and also the relation to vascular infarct territory, are displayed in Figure 2. Table 2 summarizes the comparison between patients and controls. There were no significant differences in relative flow between patients and controls. However, patients had larger interindividual flow variance in BA, P1, and P2 (Brown–Forsythe; *p* = 0.01, *p* < 0.01, and *p* = 0.02, respectively). Blood flow deviations in specific arteries are summarized in Table 3. Abnormal flow rates were identified in 20–68% of patients in the various arteries (Table 2). The most common findings were low flow in P1 and/or BA, flow asymmetries in VA or Pcom, low flow in P1 compensated by Pcom, or visualization of leptomeningeal collaterals via P2.

**TABLE 2:** Proportion of total CBF (in %) for the main arteries of the posterior circulation in patients (*n* = 25) and controls (*n* = 15).

	Controls (*n* = 15)			Patients (*n* = 25)			Comparison	
	Mean (% tCBF)	SD	10–90% PCTL	Mean (% tCBF)	SD	Outside PCTL interval, *n* (%)	Mann–Whitney *U* (p)	Brown–Forsythe (*p*)
**Total inflow**								
Posterior inflow (VA + Pcom)	28.0	3.0	23.6–32.4	26.8	7.2	11(44%)	0.36	0.02
**Individual arteries**								
VA, single (*n* = 30/50)	13.1	5.9	4.9–21.4	10.9	8.2	16(32%)	0.10	0.09
VA, R + L	26.3	3.7	20.9–32.4	21.8	8.8	11(44%)	0.09	0.10
BA	21.5	2.8	17.5–25.6	17.7	8.1	11(44%)	0.11	0.01
P1, single (*n* = 30/50)	9.0	1.9	6.0–10.9	7.1	6.2	31(63%)	0.19	0.001
P1, R + L	18.0	2.8	12.7–21.3	14.0	10.5	18(72%)	0.10	<0.01
P2, single (*n* = 30/50)	8.4	1.2	6.8–9.9	8.3	4.3	27(54%)	0.79	<0.01
P2, R + L	16.9	2.0	13.3–19.0	16.6	7.2	16(64%)	0.78	0.02
Pcom, single (*n* = 30/50)	0.9	1.8	0–5	2.4	5.5	9(18%)	0.40	0.10
Pcom, R + L	1.7	2.7	0–6.6	4.9	9.8	7(28%)	0.78	0.20
ICA, single (*n* = 30/50)	36.9	4.9	30.1–44.5	39.1	8.2	14(28%)	0.13	0.17
ICA, R + L	73.7	3.7	67.6–79.1	78.2	8.8	11(44%)	0.09	0.10

*Extreme flows are defined as being outside the 10th–90th percentile in healthy. tCBF, total cerebral blood flow; PCTL, percentile; VA, vertebral artery; Pcom, posterior communicating artery; R + L, sum of flow in right and left artery; BA, basilar artery; P1 and P2, segments of posterior cerebral artery; ICA, internal carotid artery.*

**TABLE 3:** Pathological findings indicating a hemodyrtamic disturbance in individual arteries.

	1	2	3	4	5	6	7	8	9	10	11	12	13	14	15	17	18	19	20	21	Number of patients
**Major hemodynamlc disturbance criteria**																					
BA, low flow	X	X		X	X	X	X	X		X	X	X									9
Pcom, high flow	X	X		X	X	X									X						6
P2, bilateral low flow		X								X											2
**Minor hemodynamic disturbance**																					
P1, low or reversed flow^†^	X^†^	X^†^		^†^	X	X	X	X		X	X	X		X	X		X		X		13
PI, low flow, compensated by Pcom, Normalized in P2	X				X	X	X	X							X						6
P2, unilateral low flow											X	X		X		X	X		X		6
P2, leptomeningeal collaterals									X				X		X			X		X	5
**Other findings**																					
Pcom, reversed flow																			X		1
VA:s, asymmetric or no flow	X	X	X	X	X			X	X					X	X	X	X				11
	P	P	P	P	P	P	P	P	P									P		P	

**FIGURE 3:**
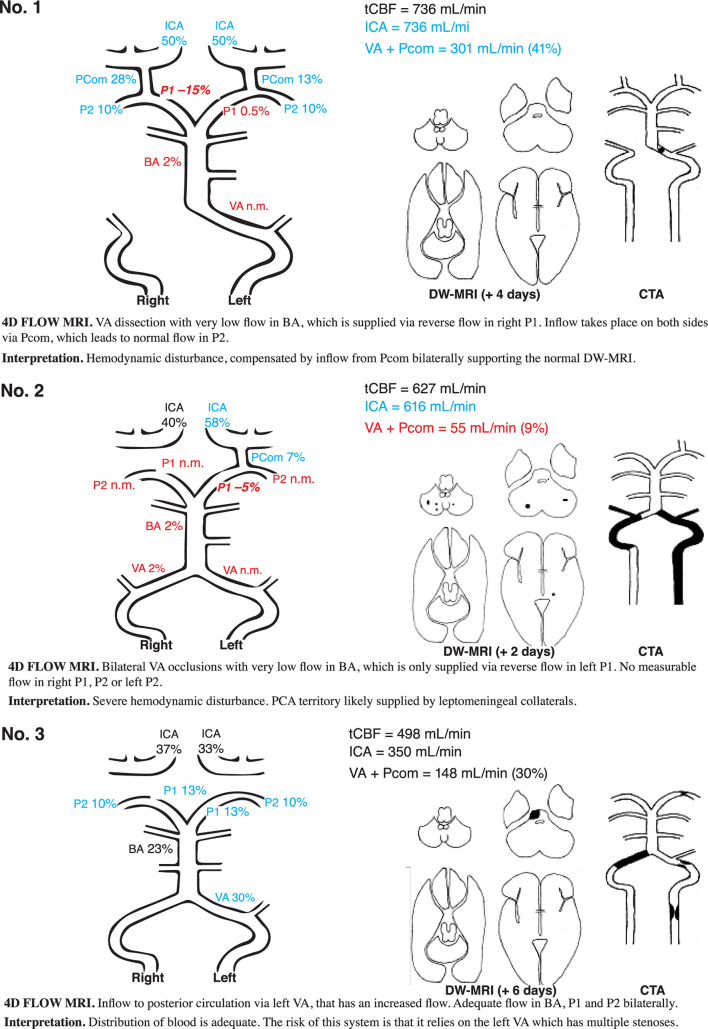
DW-MRI, CTA, and 4D flow MRI findings in three illustrative cases. Blood flow rate is presented in percent of total cerebral blood flow. Patient no. 1 had signs and symptoms corresponding to a Wallenberg syndrome and PICA infarct, however, without any DW-MRI lesion in the brainstem or cerebellum. CTA revealed a left-sided distal VA dissection. Clinical symptoms disappeared within weeks. Patient no. 2 had an acute onset of vertigo, dysarthria, and alternating left- and right-sided hemiparesis. In the aftermath, the patient had symptoms compatible with hypoperfusion (dizziness at exertion) but was deceased due to malignancy unrelated to the stroke about 1 year later. Patient no. 3 had dizziness, dysarthria, facial palsy, slight left-sided hemiparesis, and an ataxic gait disturbance. The patient did not have any neurological symptoms at follow-up. DW-MRI, diffusion-weighted magnetic resonance imaging; CTA, computed tomography angiography; blue, high flow; red low flow; black, flow within normal limits; VA, vertebral artery; BA, basilar artery; P1 and P2, segments of posterior cerebral artery; Pcom, posterior communicating artery; ICA, internal carotid artery; n.m., visible artery but flow not measurable; PICA, posterior inferior cerebellar artery.

**FIGURE 4:**
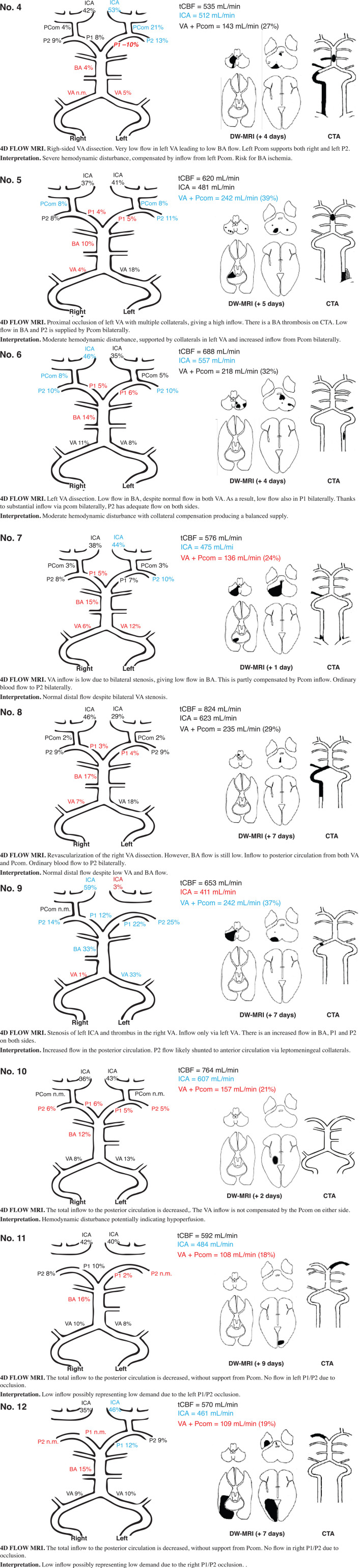
Findings on DW-MRI, CTA, and 4D flow MRI in nine selected patients (patients no. 4-12). DW-MRI, diffusion weighted magnetic resonance imaging; CTA, computed tomography angiography; blue, high flow; red, low flow; black, flow within normal limits; VA, vertebral artery; BA, basilar artery; P1 and P2, segments of posterior cerebral artery; Pcom, posterior communicating artery; ICA, internal carotid artery; n.m., visible artery but flow not measurable.

*Numbers relate to the corresponding patient number in Figures 3, 4. Patients nos. 16 and 22–25 with normal flow. VA, vertebral artery; P1 and P2, segments of posterior cerebral artery; Pcom, posterior communicating artery; CTA, computed tomography angiography; P, proximal/middle territory infarct.*

*^†^reversed flow.*

### Thrombolysis

There was a difference in relative BA flow between patients who received tPA and patients who did not receive tPA and controls [median (IQR), 22.7 (20.2–24.7) vs. 18.1 (13.5–23.3) vs. 21.1 (19.0–24.2), *p* = 0.02] where only the difference between no tPA and controls was significant in pairwise testing (*p* = 0.04 after Bonferroni correction). A difference was also found in relative P1 flow [median (IQR), 21.5 (15.3–25.4) vs. 12.0 (7.2–18.6) vs. 18.6 (16.4–20.2), *p* = 0.004] where patients who did not receive tPA had lower flow compared to both patients who did and controls (*p* = 0.03, 0.02, respectively, after Bonferroni correction).

### Correlations Between Flow in Various Arteries

In patients, there was a correlation between relative flow in the VAs and the BA, and the P1 segment of PCA (Table 4, *R* = 0.88, 0.79, both *p* < 0.001). There was a negative correlation between VA flow and Pcom flow (*R* = -0.47, *p* = 0.02). Generally, the corresponding correlations for the control group had similar trends but were weaker.

**TABLE 4:** Matrix of correlation coefficients (*r*) in patients.

	BA	P1	P2	Pcom
VA	0.88* (0.46)	0.79* (0.58*)	0.28 (−0.09)	−0.47* (−0.68*)
BA		0.90* (0.67*)	0.29 (0.28)	−0.57* (−0.74*)
P1			0.32 (0.43)	−0.57* (−0.76*)
P2				0.36 (−0.11)

Corresponding correlations for controls are separated by parentheses. VA, vertebral arteries; BA, basilar artery; Pcom, posterior communicating arteries; P1 and P2, segments of posterior cerebral artery. *p < 0.05.

### Comprehensive Assessment of Flow Patterns

Out of the nine patients with proximal/middle territory infarcts and corresponding CTA findings, five patients fulfilled the criteria for major hemodynamic disturbance. They are described in detail in Figures 3, 4 (patients nos. 1, 2, 4, 5, and 6). They were characterized by low flow in VA, BA, and P1. This was compensated by high inflows via Pcom and in all but one (Figure 3, patient no. 2); flow was normalized in P2. Three of these patients had minor hemodynamic disturbances: patient nos. 6 and 7 (Figure 4) had reduced BA and P1 flow but normal Pcom and P2 flows. Patient no. 9 (Figure 4) had severe carotid artery stenosis, no Pcom flow, but increased bilateral P2 flow, indicating leptomeningeal collaterals supplying the anterior circulation (Table 3). Patient no. 3 (Figure 3) had normal to high flows despite severe atherosclerotic steno-occlusive disease. Patients with proximal/middle territory infarcts without CTA findings had minor hemodynamic disturbances [Table 3 (nos. 19 and 21, not included in figures)] or normal 4D flow MRI findings [Table 3 (no. 16, 22–25 not included in figures)].

**FIGURE 5:**
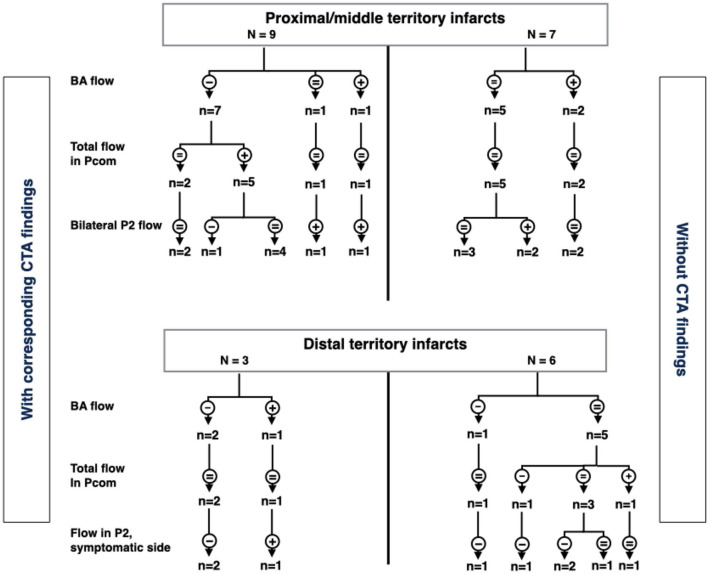
Summary of arterial flow in patients with proximal/middle territory or distal territory infarcts. CTA stenoses/occlusions are on left and right sides, respectively. The flowchart is based on flow in the basilar artery, posterior communicating arteries, and distal parts of the posterior cerebral arteries (P2). -, low flow; +, high flow; =, flow within normal limits; VA, vertebral arteries; Pcom, posterior communicating arteries; P2, second segment of posterior cerebral artery; CTA, computed tomography angiography.

In patients with distal territory infarcts, one fulfilled the criteria for major hemodynamic disturbance (no. 10), while the remaining had minor disturbances (Figure 4 and Table 3). This group was primarily characterized by low flow in the symptomatic PCA, whether it was occluded on CTA or not. Three of the nine patients also had low BA flow. In general, no compensation through Pcom was observed. Figure 5 summarizes 4D flow MRI data in relation to CTA.

## Discussion

In this feasibility study, we investigated if 4D flow MRI would be able to detect hemodynamic disturbances in consecutive patients with ischemic stroke in the VB circulation and whether these quantitative flow measurements would provide additional information regarding the patients’ hemodynamic status when added to CTA. The VB circulation of 25 patients with ischemic stroke in this territory and 15 healthy controls was described in detail. Our main finding was that in patients with proximal/middle territory infarcts and CTA findings indicative of VA steno-occlusive disease or dissection (*n* = 9), 4D flow MRI showed that five patients (56%) had major hemodynamic disturbances according to our criteria, while four (44%) did not. Among the remaining patients, only one (6%) had major hemodynamic disturbances.

This is the first study to attempt 4D flow MRI quantitative flow measurements in patients with VB ischemic stroke. However, Amin-Hanjani et al. used 2D PCMRI in patients with symptomatic, bilateral VB steno-occlusive disease in the VERiTAS study. They found that patients with low distal flow, defined as low flow in BA and P2, had significantly higher risk of new cerebrovascular events compared to patients with normal distal flow status (Amin-Hanjani et al., 2016). Romano et al. recently used the same 2D PCMRI technique in The Mechanisms of Early Recurrence in Intracranial Atherosclerotic Disease study in patients with symptomatic intracranial stenoses in both the anterior and posterior circulation. Low flow across the stenosis could not predict stroke/TIA recurrence with this more inclusive approach (Romano et al., 2021). It is not entirely straightforward to compare our data to these studies, as we present a more heterogeneous sample including any patient with VB stroke. Although a quite limited sample size, we note that among our patients with bilateral VA or BA steno-occlusive disease (Figure 3, patients nos. 2 and 3; Figure 4, patient nos. 5 and 7), one (no. 2, 25%) had low flow in both BA and P2 bilaterally, which is similar to the rates found in VERiTAS (25%). Another (Figure 4, no. 5) relied on compensatory high flow in Pcom bilaterally, while the remaining two had normal distal flow. It has not been studied whether reliance on Pcom modifies the risk of recurrent stroke either in VB atherosclerotic steno-occlusive disease or with other etiologies of stenosis and occlusion such as VA dissection. There is limited evidence to suggest fetal-type PCA (i.e., congenital reliance on Pcom to supply P2) could be associated with an increased risk of stroke (Arjal et al., 2014), but the data are conflicting (de Monye et al., 2008; Shaban et al., 2013) and a subject for further research. It seems reasonable to attempt secondary preventative endovascular intervention such as stenting or submaximal balloon angioplasty in patients with atherosclerotic steno-occlusive disease in VA or BA and low distal flow. Available data from VERiTAS suggests that patients with normal distal flow should receive best medical treatment but no endovascular intervention for secondary prevention (Amin-Hanjani et al., 2016).

Multiple observational studies have found that in patients undergoing mechanical thrombectomy for BA occlusion, the presence of Pcom is a predictor of good outcome (Goyal et al., 2016; van der Hoeven et al., 2016; Maus et al., 2019; Mahmoudi et al., 2020). Three of our patients had BA occlusions (Figure 4, nos. 4 and 5) or occlusion of a dominant VA (Figure 3, no. 1). All had markedly reduced BA flow but compensated through high Pcom flows and in two cases (nos. 1 and 4), unilateral reversed P1 flow. DW-MRI changes were, if any, limited to the cerebellum. Detailed quantitative measurements of this primary collateralization pathway have not been previously published.

We also found low posterior circulation inflow in several patients with distal territory infarcts [Figure 4, nos. 10, 11, 12 (and 16 and 18, not included in figure)]. These patients also saw a shift of flow from the posterior to the anterior circulation with supranormal flows in ICA. This has not been previously described, and it is unclear what it represents. We speculate that low posterior circulation flow may be due to reduced volumes of perfused tissue or due to altered pressure environments in the setting of acute occlusions. It is also unknown if the excess flow to the anterior circulation is shunted to the posterior circulation through peripheral pathways that we have not measured or if it represents a state of hyperperfusion.

A single patient with a distal territory infarct (no. 20, not included in figures) displayed posteroanterior flow through the ipsilateral Pcom. In a cross-sectional study, Jongen et al. investigated patients with occipital lobe infarcts and controls using 2D PCMRI. They found ipsilateral posteroanterior Pcom flow in 5/51 affected hemispheres and 4/43 unaffected hemispheres among patients (four patients had bilateral occipital infarcts). In controls, posteroanterior Pcom flow was found in 14/100 hemispheres (Jongen et al., 2004). Based on this it is possible that in our patient, this finding was also present before the vascular event.

Patients who did not receive thrombolytic treatment had lower relative BA and P1 flow compared to controls and also lower P1 flow compared to patients who did receive thrombolytic treatment. As we have shown above, it is common for patients with posterior circulation stroke to have reduced flow in these vessels, likely explaining the finding. Additionally, the NIH Natural History of Stroke study found that as many as 43% of patients treated with tPA displayed early reperfusion on renewed perfusion scans 2 h posttreatment (Majidi et al., 2019). This would explain why patients who received thrombolytic treatment more closely resembled the controls. This finding is, however, quite susceptible to confounding, as this study is small; only five patients were treated. The data are also observational, and firm causal conclusions should therefore be avoided.

The main strength of this study is the comprehensive evaluation of each case using a combination of CTA, DW-MRI, and 4D flow MRI allowing for better understanding of each cases individual flow profile. Prospective recruitment of consecutive cases with varying pathologies confirms the feasibility of using 4D flow MRI in a clinical setting. This study also has several limitations that need to be addressed. First and foremost, the small and heterogeneous sample makes drawing any conclusions relating imaging findings to outcomes very difficult. Thus, this work cannot speak to how these patients should be managed clinically. We have instead described each case on an individual basis and related flow findings to anatomy and pathology found with CTA and DW-MRI. Further studies should focus on relating 4D flow MRI findings to outcomes such as functional outcome and event-free survival. We were unable to include bedridden patients due to fire safety regulations at the research facility where the MRI was performed. This introduces a selection bias toward patients with good functional outcomes. It is possible that patients with worse functional outcomes display different flow profiles than what is presented in this study. Controls were older than patients. As older subjects generally have lower tCBF due to cerebral atrophy, this may affect reference values, causing false-positive high flows and low flows to be falsely categorized as normal. However, the distribution of flow does not seem to be affected by aging (Zarrinkoob et al., 2015), which is why we chose to mainly use relative flow for comparing patients and controls. There was a temporal delay between CTA, usually performed on admittance to the stroke unit, and MRI, performed on median 4 days after the presenting event. In combination with reperfusion therapy sometimes being employed between modalities, flow conditions may have changed. On the technical side, VENC 110 and VENC 40 were performed in series. Using both, as we have, to create a single flow profile means, measurements in the same profile will have come from different points in time, 9 min apart. If the subject’s physiology, mainly heart rate and/or blood pressure, changes, this may cause inconsistencies. Nevertheless, dual VENC is advantageous, as flows vary greatly in the VB circulation, especially if there is vascular pathology. A general limitation of 4D flow MRI is that it cannot predict flow outcomes after endovascular procedures. For example, if considering therapeutic sacrifice of a VA or BA, a balloon occlusion test in conjunction with transcranial Doppler or digital subtraction angiography (Sorteberg et al., 2008) is more appropriate to predict flow postprocedure. 4D flow MRI may instead be used to avoid invasive procedures in patients with normal flow despite structural pathology such as stenoses or dissections.

## Conclusion

4D flow MRI was able to detect flow deviations corresponding to CTA and DW-MRI findings. In addition, among patients with VA and BA pathologies on CTA, 4D flow MRI was able to distinguish patients with hemodynamic disturbances from those with normal flow profiles. We thus conclude that 4D flow MRI could add valuable hemodynamic information when used in conjunction with CTA.

## Data Availability Statement

The original contributions presented in the study are included in the article/supplementary material, further inquiries can be directed to the corresponding author/s.

## Ethics Statement

The studies involving human participants were reviewed and approved by Etikprövningsnämnden, Umeå Universitet. The patients/participants provided their written informed consent to participate in this study.

## Author Contributions

JM, JB, and AE: design and conceptualized study, analyzed the data, and drafted the manuscript of intellectual content. LZ: major role in the acquisition of data, analyzed the data, and revised the manuscript for intellectual content. AW: major role in the acquisition of MRI data, interpreted the data, and revised the manuscript for intellectual content. All authors contributed to the article and approved the submitted version.

## Conflict of Interest

The authors declare that the research was conducted in the absence of any commercial or financial relationships that could be construed as a potential conflict of interest.

## Publisher’s Note

All claims expressed in this article are solely those of the authors and do not necessarily represent those of their affiliated organizations, or those of the publisher, the editors and the reviewers. Any product that may be evaluated in this article, or claim that may be made by its manufacturer, is not guaranteed or endorsed by the publisher.
